# Breast Milk Iodine Concentration (BMIC) as a Biomarker of Iodine Status in Lactating Women and Children <2 Years of Age: A Systematic Review

**DOI:** 10.3390/nu14091691

**Published:** 2022-04-19

**Authors:** Shuchang Liu, Andrew Sharp, Elmer Villanueva, Zheng Feei Ma

**Affiliations:** 1 Department of Health and Environmental Sciences, Xi’an Jiaotong-Liverpool University, Suzhou 215123, China; shuchang.liu20@student.xjtlu.edu.cn (S.L.); elmer.villanueva@xjtlu.edu.cn (E.V.); 2 Harris-Wellbeing Research Centre, Faculty of Health & Life Sciences, University of Liverpool, Liverpool L8 7SS, UK; a.sharp@liverpool.ac.uk

**Keywords:** breast milk iodine concentration, breast milk, lactation, maternal, infant

## Abstract

Background: Iodine is needed for the production of thyroid hormones, which are essential for infant growth and development. Given that there are wide variations in breast milk iodine concentration (BMIC) and urinary iodine concentration (UIC), it is unclear if BMIC is associated with UIC in populations residing in iodine sufficient or deficient areas. Aim: To investigate if BMIC can be used as a biomarker for iodine status in lactating women and children <2 years of age. Methods: Electronic databases; PubMed, Web of Science and Scopus were searched until year 2021, for studies investigating the relationship between BMIC and UIC. Studies were reviewed for eligibility, according to inclusion and exclusion criteria, followed by data extraction, according to the PRISMA guidelines. Results: Overall, 51 studies met the criteria for inclusion in the systematic review. BMIC ranged from 18 to 1153 µg/L. In iodine-deficient and iodine-sufficient lactating women, BMIC ranged from 26 to 185 µg/L and 15 to 1006 µg/L, respectively. In most studies, the categorisation of iodine status assessed by median UIC was consistent with the categorisation of iodine status assessed by median BMIC cut off of ≥100 µg/L, to determine iodine sufficiency in lactating women and children <2 years of age. Conclusions: The systematic review indicated that BMIC is a promising biomarker of iodine status in lactating women and children <2 years of age. However, these data need to be interpreted cautiously, given the study limitations in the included studies. Future studies should consider investigating the optimal median BMIC, as there is a lack of high-quality observational and intervention studies in lactating women and infants.

## 1. Introduction

Iodine is needed by the thyroid gland to produce thyroid hormones [1]. Thyroid hormones have several important functions in the human body, including maintaining thyroid function and body metabolism [2]. Iodine deficiency is one of the most common micronutrient deficiencies worldwide, affecting 30% of the population. During pregnancy, the dietary requirement for iodine increases by 50% (i.e., 250 µg/d), because of the increased production of thyroid hormones, required for both pregnant women and their fetus [3]. The prevalence of iodine deficiency in pregnant women has been reported to range between 16.1 and 84.0% [4]. When pregnant women have insufficient iodine intake (below the recommended iodine intake), the thyroid gland is unable to produce sufficient amounts of thyroid hormones [3]. As a result, low levels of thyroid hormones can cause a number of adverse effects, particularly on brain growth and development, which are collectively known as iodine deficiency disorders (IDD) [5].

Currently, median urinary iodine concentration (UIC) is the recommended biomarker of iodine status in populations [5,6]. However, UIC only measures recent dietary iodine intake and has high intra- and inter-individual variation. WHO/ICCIDD/UNICEF have proposed a median UIC cut off of ≥100 µg/L, to indicate adequate iodine status in lactating women, despite having the same iodine intake requirement as pregnant women [5]. This is because iodine is excreted in the breast milk of lactating women. In lactating women and breast-fed infants, breast milk iodine concentration (BMIC) has been proposed to be a better biomarker of iodine status. Studies have reported that in iodine-sufficient areas (as indicated by median infant UIC and adults ≥100 µg/L), median BMIC ranged between 150 and 180 µg/L [7,8,9]. Therefore, if pregnant or lactating women are iodine deficient, their infants may be at risk of iodine deficiency, which can lead to increased risk of developing cognitive and psychomotor impairments [10]. This is because infants are sensitive to maternal iodine intake. Exclusively breast-fed infants depend entirely on their mother’s BMIC for thyroid hormone synthesis, because they do not have considerable thyroxine stores compared with adults [11].

One of the research priorities recommended by the World Health Organization (WHO) is the need for more studies measuring BMIC [12]. Since there are wide variations in BMIC, it is unclear if BMIC is associated with the UIC of lactating women and children <2 years of age, residing in iodine-sufficient or -deficient regions. In addition, there are no accepted BMIC cut offs to categorise iodine sufficiency in lactating women and children <2 years of age. Therefore, the systematic review will firstly report the analytical methods used to measure BMIC and UIC, followed by observational studies and intervention studies, measuring both the BMIC and UIC of lactating women and children <2 years of age.

## 2. Methods

The systematic review was conducted in accordance with the Preferred Reporting Items for Systematic Reviews and Meta-analyses (PRISMA) guidelines [13]. The full protocol of the systematic review was registered at PROSPERO (at https://www.crd.york.ac.uk/prospero/ (accessed on 13 October 2021), as CRD42021231711. The systematic review does not contain any studies with human participants or animals performed by any of the authors listed and is entirely based on previously conducted studies.

### 2.1. Search Strategy and Selection Criteria

The literature reporting results from studies examining the relationship between BMIC and UIC were reviewed. Three online electronic databases (PubMed, Web of Science and Scopus) were used to do the selection of articles until year 2021. Other relevant databases and search engines including Cochrane Library CENTRAL and Google Scholar were also searched. In addition, additional studies from references were located in the retrieved articles.

The major key search terms used included: “iodine”, “milk” and “urine”. Other term combinations were used as the searching strategy, such as the term ‘BMIC’ or ‘breast milk iodine concentration’ plus ‘UIC’ or ‘urinary iodine concentration’ plus related terms ‘maternal iodine status’, ‘human milk’, ‘colostrum’, ‘mature milk’, ‘lactation’, ‘lactating’ ‘postpartum’, ‘infants’, ‘newborns’, ‘offspring’ and ‘neonates’. The two databases PubMed and Web of Science can be found on the Endnote website, so the searching results from the third database Scopus were imported into the Endnote website to do the screening. Duplications in the primarily articles were removed between all databases. Title screening was then performed to exclude articles not relevant to BMIC and UIC by title. The inclusion and exclusion criteria were applied to screen the abstracts and full texts of the remaining articles.

Inclusion criteria were: original articles published until year 2021; exposures of the study most relevant to maternal iodine status; outcomes of the study must report BMIC and urinary iodine (UIC and/or I/Cr ratio); studies must focus on healthy women; studies must have either one of the following: BMIC and urinary iodine (UIC and/or I/Cr ratio) of lactating women, BMIC of lactating women and urinary iodine (UIC and/or I/Cr ratio) of children <2 years of age [5]; studies must be reported in the English language. For the purpose of the systematic review, the term ‘children’ was used to include neonates, infants and toddlers <2 years of age where appropriate. Exclusion criteria were: studies in animals; exposures of the study are not relevant to maternal iodine status; exposures of the study focus on not only iodine; outcomes of the study are not relevant to BMIC and UIC; studies reported just on single case (e.g., letters and case reports); reviews, rather than original research articles.

### 2.2. Data Extraction

Two investigators (SL and ZFM) independently extracted the following data from the selected studies included in the systematic review: the name of the first author, the type of the study, the year of the study published, the setting of the study, sample size, the characteristics of participants (including age and gestation weeks), and primary outcomes (i.e., BMIC and UIC), secondary outcomes (including clinical outcomes). Where necessary, further data or explanation of data analyses was sought from the authors of the studies. There was a high agreement between both investigators (SL and ZFM). Any identified discrepancies were discussed and resolved by consensus. The following data were extracted: first author, year of publication, country or location of study, study design, number of mothers and infants, infant age or time of postpartum, median or mean BMIC and UIC data of lactating women or infants, methods to assess BMIC and UIC.

### 2.3. Quality Assessment

Two scales were used to assess the quality of these studies depending on the types of studies. The Jadad scale was used to evaluate the quality of the randomised clinical trials, while the Newcastle–Ottawa scale was used to evaluate the quality of non-randomised and observational studies.

Quality of interventional studies was assessed using the Jadad scale [14], using a five-point checklist with yes/no answers to questions relating to methodology (Supplementary Tables S1–S3). This is a proven report quality indicator which is specifically designed for randomised intervention studies [14]. Points could be earned if the study was described as randomised (+1); the method of randomisation was described, and appropriate (+1); the study was performed double-blind (+1); the method of blinding was described and appropriate (+1); there was a description of withdrawals and subject dropouts (+1) [14]. There was no point awarded if the study was not described as randomised (+0); if the study was not performed double-blind (+0); if there was no description of withdrawals and subject dropouts (+0). Points were deducted if the method of randomisation was described and was inappropriate (−1); if the method of blinding was described and was inappropriate (−1) [14].

An adapted version of the Newcastle–Ottawa scale was used to assess the quality of the cohort studies [15]. The Newcastle–Ottawa scale has been recommended for assessing the quality of non-randomised studies, categorized into three dimensions including (1) selection, (2) comparability, and (3) assessment [16]. High-quality characteristics within each item according to these three dimensions were awarded a star, a maximum of five stars was awarded for selection, a maximum of four stars was awarded for comparability and a maximum of four stars was awarded for assessment [15]. A good point of this assessment tool is the avoidance of reporting the summary scores, which is difficult to interpret and can be considered unreliable [17].

The study quality was assessed using the criteria in Supplementary Tables S2 and S3. For descriptive purposes, scores of 0–4, 5–8, and 9–13 were used to indicate a low, moderate and high quality, respectively, for Newcastle–Ottawa scale articles. For Jadad scale articles, scores of 0–1, 2–3, and 4–5 were used to indicate a low, moderate and high quality, respectively.

### 2.4. Definitions and Outcomes

Currently, to our knowledge, there is no consensus regarding the suitable BMIC cut off indicative of iodine sufficiency in lactating women. Therefore, for the purpose of the systematic review, a median BMIC cut off of ≥100 µg/L was used to determine iodine sufficiency. This is because a full-term infant is considered to need 15 µg iodine/kg/day for maintaining normal thyroid metabolism [7,18,19]. A median UIC cut off ≥100 µg/L is used to define adequate iodine intake in lactating women and children <2 years of age [5].

## 3. Results

**Figure 1:**
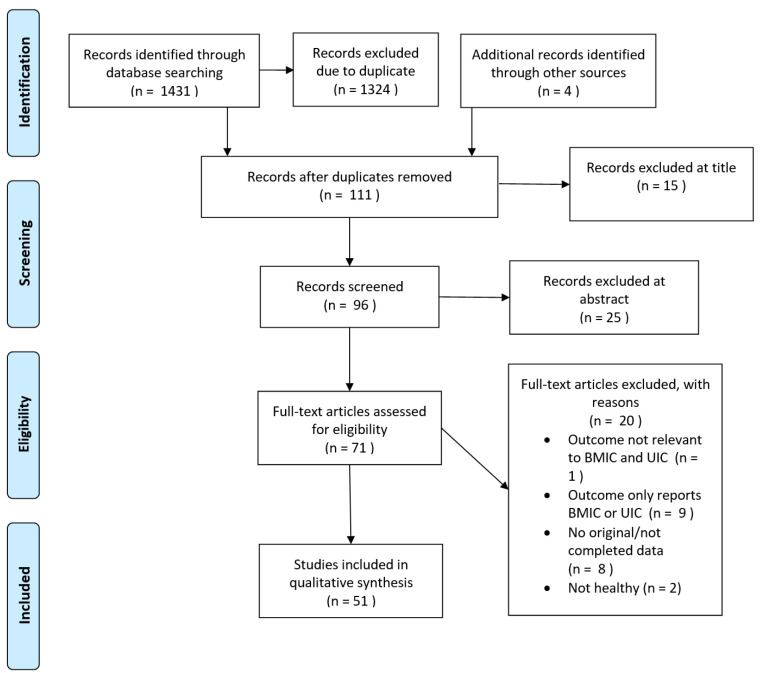
PRISMA 2009 Flow Diagram. Adapted from Moher et al. [13].

Figure 1 shows the flow chart for this review. In total, 1431 papers were identified in three online electronic databases (PubMed, Web of Science and Scopus). After removing duplications and adding 4 articles from the other sources (e.g., references list or online), 111 appeared potentially relevant. Then, the title and abstract screening were conducted according to the inclusion and exclusion criteria. Of these, 71 publications appeared potentially relevant and were assessed as full-text papers for inclusion. There were 20 studies that were excluded due to the lack of inclusion criteria; of these, most (*n* = 9) failed to report data of both BMIC and UIC, followed by the reason of not original/completed data paper (*n* = 8), not healthy women (*n* = 2) and not relevant to BMIC and UIC (*n* = 1). A total of 51 studies fulfilled the inclusion criteria and were included in the present systematic review. All included articles were published between 1992 and 2021.

These 51 studies, including 29 countries on 5 continents, cover a wide geographical and socioeconomic spectrum, which could partially represent the BMIC and UIC situation in the world. The range of the sample size was from 10 to 2554. The studies were performed in China (*n* = 7), Iran (*n* = 6), Denmark (*n* = 3), Thailand (*n* = 3), Turkey (*n* = 3), New Zealand (*n* = 2), Morocco (*n* = 3), Algeria (*n* = 2), Australia (*n* = 2), Croatia (*n* = 2), South Africa (*n* = 2), United States (*n* = 2), Azerbaijan (*n* = 1), Brazil (*n* = 1), Ethiopia (*n* = 1), Gambia (*n* = 1), Germany (*n* = 1), Hungary (*n* = 1), Iceland (*n* = 1), India (*n* = 1), Italy (*n* = 1), Korea (*n* = 1), Nepal (*n* = 1), Norway (*n* = 1), Portugal (*n* = 1), Philippines (*n* = 1), Spain (*n* = 1), Switzerland (*n* = 1), and the Netherlands (*n* = 1). Of these, 50 studies focused on just one country, while only one study was performed in several countries, namely, China, Philippines, Croatia and Morocco [20]. The quality scores of the studies included in the systematic review ranged from 6 to 12 for observational or non-randomised intervention studies and 2 to 5 for randomised intervention studies (Table 1). There were 31 observational or non-randomised intervention studies and 2 randomised intervention studies that received the highest scores.

The inductively coupled plasma mass spectrometer method (ICP-MS) and Sandell–Kolthoff reaction are obviously the most commonly used methods among all the methods for detecting BMIC (98%) and UIC (98%) (Table 2). Only one study (2%) used reversed-phase high-performance liquid chromatography (HPLC). The detection methods of BMIC, ICP-MS and Sandell–Kolthoff reaction account for 47% and 51%, respectively. Of the types of biochemical methods used to assess UIC, a majority of the studies chose the Sandell–Kolthoff reaction (78%), followed by ICP-MS (20%) and HPLC (2%). The majority of studies (73%) employed the same method to assess both BMIC and UIC; only 14 studies (27%) used two different types of methods to assess BMIC and UIC. 

### 3.1. Studies Measuring Both UIC and BMIC of Lactating Women

#### BMIC Cut Off of ≥100 µg/L to Indicate Iodine Sufficiency

Thirty-eight observational studies (as indicated by the number of references), measuring both the UIC and BMIC of lactating women, were identified (Table 3). Fourteen studies reported that iodine-deficient lactating women (median UIC < 100 µg/L) had a median BMIC <100 µg/L, while only 4 reported that iodine-deficient lactating women (median UIC <100 µg/L) had a median BMIC ≥ 100 µg/L. On the other hand, 16 studies reported that iodine-sufficient lactating women (median UIC ≥ 100 µg/L) had a median BMIC ≥ 100 µg/L, while 7 studies reported that iodine-sufficient lactating women (median UIC ≥ 100 µg/L) had a median BMIC < 100 µg/L.

Six intervention studies, measuring both the UIC and BMIC of lactating women, were identified (Table 3). The longest duration of invention was 9 months. In a study by Bouhouch et al., despite the supplementation of one dose of 400 mg iodine as oral iodised oil, the women remained iodine deficient (both UIC and BMIC) throughout the intervention period. Although the study by Eriksen et al. supplemented women with 300 µg iodine, containing a prenatal multiple micronutrient supplement, the median BMIC of lactating women was <100 µg/L at 12 weeks postpartum. Another study, by Gebreegziabher et al., reported that the median BMIC values of women either receiving 225 µg iodine as a potassium iodide capsule daily or 450 g of iodized salt (30–40 µg iodine as KIO_3_/g of salt) weekly for 6 months was ≥100 µg/L at 6 months postpartum. The study by Nazeri et al. reported that both median BMIC values of women receiving iodine-fortified milk and control group were ≥100 µg/L at 1 month postpartum; women receiving iodine-fortified milk had a significantly higher median UIC than the control group (*p* < 0.001). The study by Stoutjesdijk et al. reported that Dutch women supplemented with 150 μg iodine at 20 weeks of gestation had both median BMIC and UIC values ≥ 100 µg/L at 4th week of postpartum, indicating iodine sufficiency. A study by Sukkhojaiwaratkul et al. reported that, despite the fact that both women receiving 200 µg iodine table daily and women in the non-supplemented group were iodine sufficient at 2 months postpartum, the median BMIC of women in the supplemented group was higher than the non-supplemented group, suggesting the importance of maternal iodine supplementation in the improving iodine status of breast-fed infants.

### 3.2. Studies Measuring UIC of Infants and BMIC of Lactating Women

#### BMIC Cut Off of ≥100 µg/L to Indicate Iodine Sufficiency

Twenty-nine observational studies (as indicated by the number of references), measuring both the UIC of infants and BMIC of lactating women, were identified (Table 4). Eight studies reported iodine-deficient infants (median UIC < 100 µg/L) born to lactating women with a median BMIC < 100 µg/L, while no studies reported iodine-deficient infants (median UIC < 100 µg/L) born to lactating women with a median BMIC ≥ 100 µg/L. On the other hand, 19 studies reported iodine-sufficient infants (median UIC ≥ 100 µg/L) born to lactating women with a median BMIC ≥ 100 µg/L, while 3 studies reported iodine-sufficient infants (median UIC ≥ 100 µg/L) born to lactating women with a median BMIC < 100 µg/L.

Six intervention studies, measuring both the UIC of infants and BMIC of lactating women, were identified (Table 4). The longest duration of infants supplemented with iodine was 9 months. One study, by Bouhouch et al., reported that at 6 months, infants were iodine sufficient based on infant UIC, but iodine deficient based on BMIC. Three months later, infant UIC decreased to <100 µg/L and BMIC remained at <100 µg/L. Another study, by Gebreegziabher et al., reported that the median UIC of infants born to women either receiving 225 µg iodine as a potassium iodide capsule daily or 450 g of iodized salt (30–40 µg iodine as KIO_3_/g of salt) weekly for 6 months was ≥100 µg/L at 6 months postpartum. A study by Gutierrez-Repiso et al. reported that both women and their infants in the 300 and control group were iodine sufficient, based on median BMIC and infant UIC values (≥100 µg/L). A study by Kirk et al. demonstrated that, although median infant UIC was ≥100 µg/L, single-dose iodine supplements were not effective in improving BMIC values. Another study, by Nøhr et al., reported that both groups, one receiving tablets containing iodine and one not receiving iodine supplementation, had both median BMIC and UIC values < 100 µg/L. A study by Nazeri et al. reported that both median BMIC values of women and infants in the iodine-fortified milk and control groups were ≥100 µg/L at 1 month postpartum.

## 4. Discussion

The systematic review reveals that BMIC ranged from 26 to 185 µg/L and 15 to 1006 µg/L in iodine-deficient and iodine-sufficient lactating women, respectively. Only few studies on BMIC were from excessive iodine areas (median UIC ≥ 300 µg/L based on non-pregnant adult populations). The majority of the studies on BMIC findings were conducted in both iodine-deficiency and iodine-sufficiency areas. However, most studies were cross-sectional studies and did not clearly indicate if the infants were breast-fed. The dose of iodine supplementation ranged between 30 µg and 400 mg iodine. In terms of the dose of iodine that the infants received, there was a variation in the duration of iodine supplementation, the amount and form of the iodine supplemented to the lactating women. Therefore, high-quality data on the BMIC and UIC of lactating women, with different iodine status (iodine deficiency, iodine sufficiency, and excessive iodine) with breast-fed infants, are generally lacking. 

Over the past two decades, Sandell–Kolthoff reaction, which is a traditional colorimetric method, has been commonly used to detect UIC. One of the possible reasons is because, according to the WHO/ICCIDD/UNICEF, Sandell–Kolthoff reaction, using ammonium persulfate as the digestion method, has been recommended, which is also known as method A [5]. There is also another method, called method B; the only difference is the digestion step, as method A uses the ammonium persulfate to digest urine samples, while method B digests with chloric acid [5]. However, chloric acid has potential hazards and it is more toxic as the digestant, so using ammonium persulfate is currently recommended by WHO/ICCIDD/UNICEF, and the method has been modified from the previous method [5,71]. The current recommended Sandell–Kolthoff reaction has simple, convenient and economic advantages [5].

Currently, there are no recommended methods for analysing BMIC. Spectrophotometric Sandell–Kolthoff, HPLC, and ICP-MS-based methods have been used to measure BMIC. Therefore, one of the challenges in comparing BMIC across different studies is due to the differences in the analytical methods used and lack of method standardisation across different analytical methods [72]. In addition, studies on BMIC were based on spot samples at different sampling times and stages of lactation. Therefore, the inconsistencies in these findings on nutrient compositions may be due to the different stages of lactation, sampling time, time of the day, maternal iodine status, and individual variation. However, BMIC does not seem to be affected by the sampling methods (i.e., time of day, before or after the lactation session, and left or right breast) [23,73]. 

### 4.1. Factors Influencing BMIC

BMIC may be affected by physiological fluctuations [74]. For example, median BMIC gradually increases with time, from birth up to 12 months [37]. A study by Etling et al. reported that BMIC was reported to increase during the first month of the lactation period [75]. Another study reported that BMIC decreased during the first 6 months of the lactation period [18], which may be due to suboptimal iodine status in lactating women. However, another study reported that BMIC varied from day to day [76]. These inconsistent findings should be confirmed in larger longitudinal studies of lactating women.

Several studies have reported that the nutrient content of breast milk differs significantly between different stages of the lactation period, suggesting that BMIC follows a similar pattern to that of other nutrients [77]. Higher BMIC has been observed during the first few days of the lactation period, followed by a decreasing trend over time, which may be because the colostrum is gradually changed into mature breast milk. However, there is no difference in BMIC between colostrum and mature breast milk in iodine-deficient lactating women [48]. 

### 4.2. BMIC as a Biomarker to Assess Iodine Status in Lactating Women and Children <2 Years of Age

A UIC cut off of ≥100 has been proposed to indicate iodine sufficiency in children aged <2 years [5]. Iodine intake of breast-fed infants corresponds to BMIC, because the dietary iodine sources of breast-fed infants depend entirely on the mothers’ iodine intake. Therefore, BMIC is also an important biomarker of iodine status for breast-fed infants [20]. In non-lactating women, absorbed iodine is partly transported to the thyroid gland and the remaining iodine (~90%) is cleared by passive renal glomerular filtration [78]. However, in lactating women, absorbed iodine is also transported to the mammary gland by NIS (sodium iodide symporter) [78]. Therefore, UIC is consequently lower, and the median UIC, indicating iodine sufficiency in lactating women (who are breastfeeding), is similar to non-pregnant individuals (≥100 µg/L), although lactating women (who are breastfeeding) have higher iodine requirements [5,7]. In iodine-sufficient lactating women (median UIC ≥100 µg/L), higher fractional iodine is excreted into breast milk at a lower range of daily maternal iodine intake and, consequently, renal fractional iodine excretion is decreased [20]. Even in non-lactating women, lower UIC is reported during lactation than in pregnancy, which might be due to the higher clearance of circulating iodine to the restoration of the depleted thyroid gland for the restoration of the depleted thyroid gland [79]. Therefore, BMIC is considered a more reliable biomarker of iodine status in lactating women than UIC.

However, there have been some studies reporting discrepancies between the UIC of lactating women and their BMIC, suggesting that BMIC may not be able to accurately reflect infant iodine status. Therefore, there is a need to further explore the reliability of BMIC as a biomarker of iodine status in infants. 

### 4.3. Why Did Some Lactating Women Classified as Iodine Sufficient by UIC Have a BMIC Less Than the Proposed BMIC Cut Offs (i.e., BMIC Considered Iodine Deficient)?

This is probably as a result of the recent maternal dietary iodine intake and duration of the lactation period [36,74,80]. Future studies are needed to investigate other factors, such as the genetic variations in the SLC_5_A_5_ gene in relation to BMIC, which has been reported to play an important role in the iodine transfer into breast milk [81].

### 4.4. Why Did Some Lactating Women Classified as Iodine Deficient by UIC Have a BMIC Equivalent or Higher Than the Proposed BMIC Cut Offs (i.e., BMIC Considered Iodine Sufficient)?

In the systematic review, some studies reported that iodine-deficient lactating women (median UIC < 100 µg/L) had a median BMIC ≥ 100 µg/L. In iodine-deficient regions, since the mammary gland can concentrate iodine, iodine supply to the infants may be maintained via breast milk, even if the mothers are iodine deficient [8,62]. This may help explain why, in iodine-deficient regions, lactating women were classified as iodine deficient based on median UIC, but were iodine sufficient according to their median BMIC [7,8].

### 4.5. What Is an Appropriate BMIC Cut Off to Categorise Iodine Sufficiency in Lactating Women and Children <2 Years of Age?

Currently, there are no official guidelines on the median BMIC cut off to indicate iodine sufficiency in lactating women and children <2 years of age. However, there have been some median BMIC cut offs proposed to indicate iodine sufficiency (i.e., 50, 75, 80, 92, and 100 µg/L) [7,26,73,82,83]. A median BMIC cut off of >75 µg/L was suggested by Azizi and Smyth [73]; however, another higher median BMIC cut off of ≥100 µg/L has also been proposed [7,83]. This is because a full-term infant is considered to need 15 µg iodine/kg/day for maintaining normal thyroid metabolism [7,18,19]. Semba and Delange concluded that BMIC should be between 100 and 200 µg/L to meet the recommendations of the Food and Nutrition Board (FNB) of the Institute of Medicine (IoM) [7]. A study by Dold et al. suggested that, in iodine-sufficient regions, a BMIC reference range of 60–465 µg/kg can be used to suggest iodine sufficiency in lactating mothers and breast-fed infants [20]. However, it is unclear whether the similar BMIC reference range can be applied in iodine-deficient regions. 

There is a wide range of median or mean BMIC across different studies and regions [7,8,73]. In the USA, the median BMIC of lactating women ranges from 35 to 155 µg/L [84]. Several possible reasons might have contributed to this phenomenon, which include: lack of standardisation of breast milk collection methods, physiological mechanisms during pregnancy and lactation, iodine status during pre-pregnancy or pregnancy, dietary intake, and the region where the study was conducted (i.e., iodine deficient or iodine sufficient). A study by Leung et al. reported an increase in BMIC following acute maternal dietary iodine intake, suggesting that BMIC can be influenced by physiological mechanisms [74].

The main strength of the systematic review is the inclusion of the BMIC and UIC of lactating women and infants. In addition, the analytical methods of BMIC and UIC were reviewed. The limitations of the included studies were as follows: cross-sectional studies did not clearly indicate if the infants were breast-fed; further, high-quality data on the BMIC and UIC of lactating women with different iodine status with breast-fed infants are generally lacking. Concerning BMIC, differences in the analytical methods used and lack of method standardization across different analytical methods is an important limitation. Given the limited numbers of studies (*n* = 6) that have assessed BMIC across subgroups of lactating women, at different stages of the lactation period, these findings should be interpreted cautiously. Studies reporting whether BMIC changes with time of lactation period are inconsistent [8]. It is unclear if BMIC varies with regard to the collection time of the day, fore or hind milk, or left or right breast [23,59]. There is only one study, by Andersen et al., that collected breast milk samples from one and both breasts of breastfeeding women [23]. The authors reported no difference in median BMIC between one and both breasts (83 vs. 83 µg/L). In addition, breast-milk sampling, performed before or after breastfeeding, did not influence median BMIC (82 vs. 78 µg/L) [23,85]. 

In conclusion, this systematic review revealed that, although BMIC can be used to assess iodine status in lactating women and children <2 years of age, it is associated with some limitations, including an optimal BMIC cut off used to indicate iodine sufficiency. Therefore, it is difficult and challenging to draw a firm conclusion regarding the usefulness of BMIC as a biomarker of iodine status based on these studies. More well-designed, large-scale studies are needed to examine the usefulness and feasibility of BMIC in assessing iodine status in lactating women and children <2 years of age, with different levels of iodine intake.

## Figures and Tables

**Table 1:** Summary of the association between BMIC and UIC and study scores for quality criteria.

*Reference*	*Adapted Newcastle–Ottawa Scale*				
	*Association between BMIC and UIC*	*Selection* (Maximum 5 *)	*Comparability* (Maximum 4 *)	*Assessment* (Maximum 4 *)	*Total Scores* (Maximum 13 *) ^1^
*Observational/Non-randomised intervention studies*					
Aakre et al., 2015 [21]	+ ^2^	***	**	**	7
Aakre et al., 2016 [22]	+ ^3^	***	**	***	8
Anderson et al., 2014 [23]	+ ^2^	****	***	**	9
Anderson et al., 2010 [24]	+	****	**	**	8
Azizi, 2007 [25]	− ^2^	****		****	8
Bazrafshan et al., 2005 [26]	− ^2^	****		**	6
Böhles et al., 1993 [27]	− ^2^	****		****	8
Budak et al., 2009 [28]	+	****		****	8
Chan et al., 2003 [29]	+ ^2^	****	*	****	9
Chen et al., 2020 [30]	+	****	**	***	9
Chung et al., 2009 [31]	+ ^3^	****		***	7
Costeira et al., 2009 [32]	+	****	**	***	9
de Lima et al., 2013 [33]	+ ^3^	****	**	****	10
Dold et al., 2017 [20]	+	****	***	***	10
Dumrongwongsiri et al., 2018 [34]	+	****	***	****	11
Groufh-Jacobsen et al., 2020 [35]	+ ^2^	****	****	***	11
Gutierrez-Repiso et al., 2014 [36]	+ ^3^	****	*	****	9
Henjum et al., 2016 [37]	+ ^2^	****	*	***	8
Huynh et al., 2017 [38]	+	****	***	***	10
Isiklar Ozberk et al., 2018 [39]	+	****	**	****	10
Jin et al., 2021 [40]	+	****	**	****	10
Kart et al., 2021 [41]	+	****	***	****	11
Kirk et al., 2012 [42]	− ^3^	****	**	****	10
Kurtoglu et al., 2004 [43]	+	****	*	****	9
Laurberg et al., 2004 [44]	+ ^2^	****	**	***	9
Liu et al., 2015 [45]	+	****	**	****	10
Liu et al., 2020 [46]	+	****	*	****	9
Mobasseri et al., 2014 [47]	−	****	*	***	8
Nazeri et al., 2018 [48]	−	****	***	****	11
Nøhr et al., 1994 [49]	+ ^3^	****	*	***	8
Ordookhani et al., 2007 [50]	+	****	*	****	9
Osei et al., 2016 [51]	+	****	****	****	12
Osei et al., 2017 [52]	+	****	**	***	9
Pal et al., 2018 [53]	+	****	**	****	10
Pearce et al., 2007 [54]	+ ^2^	****	*	****	9
Petersen et al., 2020 [55]	− ^2^	****	***	****	11
Pongpaew et al., 1999 [56]	+ ^2^	****	*	****	9
Prpić et al., 2021 [57]	−	****	***	****	11
Samson et al., 2021 [58]	− ^2^	****	****	***	11
Skeaff et al., 2005 [59]	+ ^3^	****	**	***	9
Stinca et al., 2017 [60]	+	****	**	****	10
Sukkhojaiwaratkul et al., 2014 [61]	− ^2^	****		***	7
Vermiglio et al., 1992 [62]	+	****		****	8
Wang et al., 2018 [63]	+	****	**	***	9
Wang et al., 2009 [64]	+	****		****	8
Yan et al., 2005 [65]	−	****		***	7
	***Jadad Scale***				
	***The Jadad Scores (Maximum 5)***				
*Randomised interventional studies*					
Bouhouch et al., 2014 [66]	+	5			
Eriksen et al., 2020 [67]	+ ^2^	4			
Gebreegziabher et al., 2017 [68]	+	3			
Nazeri et al., 2017 [69]	−	3			
Stoutjesdijk et al., 2018 [70]	+ ^2^	2			

+, BMIC has a positive association with UIC; −, BMIC has a negative association with UIC. ^1^ The asterisk denotes the score(s) for each criterion. Each asterisk denotes a score of 1. ^2^ Association of BMIC and UIC of lactating women. ^3^ Association of BMIC and UIC of infant. Newcastle–Ottawa scale. Overall Quality Assessment Rating; High-quality characteristics within each category were awarded a star, up to a maximum of five stars for selection, four stars for comparability and four stars for assessment. Jadad scale. Overall Quality Assessment Rating; High-quality characteristics within each category were awarded 1 score, 0 or −1 score was awarded if not met the characteristics, up to a maximum of 5 scores in total.

**Table 2:** Types of BMIC and UIC methods in studies assessing BMIC and UIC in lactating women and their infants.

Studies	Year	BMIC Method	UIC Method
Vermiglio et al. [62]	1992	S-K ^2^	S-K ^2^
Böhles et al. [27]	1993	S-K ^1^	S-K ^3^
Nøhr et al. [49]	1994	S-K ^3^	S-K ^3^
Pongpaew et al. [56]	1999	S-K	S-K
Chan et al. [29]	2003	ICP-MS	ICP-MS
Kurtoglu et al. [43]	2004	HPLC	HPLC
Laurberg et al. [44]	2004	S-K	S-K
Bazrafshan et al. [26]	2005	S-K	S-K
Skeaff et al. [59]	2005	S-K	S-K
Yan et al. [65]	2005	S-K	S-K
Azizi [25]	2007	S-K	S-K
Ordookhani et al. [50]	2007	S-K	S-K
Pearce et al. [54]	2007	S-K	S-K
Budak et al. [28]	2009	S-K	S-K
Costeira et al. [32]	2009	S-K ^2^	S-K
Chung et al. [31]	2009	S-K	S-K
Wang et al. [64]	2009	S-K	S-K
Anderson et al. [24]	2010	ICP-MS	S-K
Kirk et al. [42]	2012	ICP-MS	ICP-MS
de Lima et al. [33]	2013	ICP-MS	ICP-MS
Anderson et al. [23]	2014	S-K ^3^	S-K ^3^
Bouhouch et al. [66]	2014	ICP-MS	S-K
Gutierrez-Repiso et al. [36]	2014	S-K ^3^	S-K ^3^
Mobasseri et al. [47]	2014	S-K	S-K
Sukkhojaiwaratkul et al. [61]	2014	S-K	S-K
Aakre et al. [22]	2015	ICP-MS	S-K
Liu et al. [37]	2015	S-K ^3^	S-K ^3^
Aakre et al. [22]	2016	ICP-MS	S-K
Henjum et al. [37]	2016	ICP-MS	S-K
Osei et al. [51]	2016	ICP-MS	S-K
Osei et al. [52]	2016	ICP-MS	S-K
Dold et al. [20]	2017	ICP-MS	S-K
Gebreegziabher et al. [68]	2017	ICP-MS	ICP-MS
Huynh et al. [38]	2017	ICP-MS	S-K
Nazeri et al. [69]	2017	S-K	S-K
Pal et al. [53]	2017	S-K ^3^	S-K ^3^
Stinca et al. [60]	2017	ICP-MS	S-K
Dumrongwongsiri et al. [34]	2018	ICP-MS	S-K
Isiklar Ozberk et al. [39]	2018	S-K	S-K
Nazeri et al. [48]	2018	S-K	S-K
Stoutjesdijk et al. [70]	2018	ICP-MS	ICP-MS
Wang et al. [63]	2018	ICP-MS	S-K
Chen et al. [30]	2020	ICP-MS	S-K
Eriksen et al. [67]	2020	ICP-MS	ICP-MS
Groufh-Jacobsen et al. [35]	2020	ICP-MS ^4^	ICP-MS ^4^
Petersen et al. [55]	2020	ICP-MS	ICP-MS
Liu et al. [46]	2020	S-K ^3^	S-K ^3^
Jin et al. [40]	2021	ICP-MS	ICP-MS
Kart et al. [41]	2021	S-K	S-K
Prpić et al. [57]	2021	ICP-MS	S-K
Samson et al. [58]	2021	ICP-MS	ICP-MS

S-K, Sandell–Kolthoff reaction; ICP-MS, Inductively Coupled Plasma Mass Spectrometer; HPLC, reversed-phase high-performance liquid chromatography, ^1^ acid digestion by a mixture of H_2_SO4, HClO_4_ and HNO3; ^2^ using the chloric acid digestion method, ^3^ Ce/As, arsenic–cerium catalytic spectrophotometry; ^4^ ICP-QQQ, Triple Quadruple Inductively Coupled Plasma Mass Spectrometer.

**Table 3:** BMIC and UIC of lactating women.

Author, Year	Country	Sample Size of Lactating Women	Time of Postpartum (Days/Weeks/Months)			BMIC ^1^ (µg/L)				UIC ^1^ (µg/L)			Comments
***Observational study***													
Aakre et al., 2015 [21]	Algeria	111	0–7 months			479				350			
Anderson et al., 2014 [23]	Denmark	127	31 days ^1^	Total			83	Total			72		
				Iodine-supplemented			112	Iodine-supplemented			83		
				Non-supplemented			72	Non-supplemented			65		
Anderson et al., 2010 [24]	Switzerland	507	6–12 months			49				67			
Azizi, 2007 [25]	Iran	100	NS			93				259			
Bazrafshan et al., 2005 [26]	Iran	100	30–180 days			94				259			
Böhles et al., 1993 [27]	Germany	10	5–7 days			55 ^2^				134 ^3^			Small sample size
Budak et al., 2009 [28]	Turkey	35	18 days			33 ^2^				70			
Chan et al., 2003 [29]	Australia	50	4 days			84				47			
Chen et al., 2020 [30]	China	634	1–24 weeks			165				122			
Costeira et al., 2009 [32]	Portugal	140	3 months	3 days			95	3 days			35		
				3 months			70	3 months			50		
Dold et al., 2017 [20]	China, Philippines, Croatia and Morocco	866	3 month	China (*n* = 298)			170	China (*n* = 298)			107		
				Philippines (*n* = 281)			185	Philippines (*n* = 281)			89		
				Croatia (*n* = 73)			124	Croatia (*n* = 73)			35		
				Morocco (*n* = 74)			30	Morocco (*n* = 74)			33		
Dumrongwongsiri et al., 2018 [34]	Thailand	71	NS			255				149			
Groufh-Jacobsen et al., 2020 [35]	Norway	133	1–12 months			71				80			
Henjum et al., 2016 [37]	Nepal	500	2–12 months			250				230			
Huynh et al., 2017 [38]	Australia	696	3 months			127				125			
Isiklar Ozberk et al., 2018 [39]	Turkey	107	NS			97				135			
Jin et al., 2021 [40]	New Zealand	87	3 months			69				82			
Kart et al., 2021 [41]	Turkey	334	4–6 days			138				125			
Kurtoglu et al., 2004 [43]	Turkey	70	5 days			73				30			
Laurberg et al., 2004 [44]	Denmark	140	5 days	Smokers (*n* = 50)			26 ^2^	Smokers (*n* = 50)			41 ^2^		
				Non-smokers (*n* = 90)			54 ^2^	Non-smokers (*n* = 90)			40 ^2^		
Liu et al., 2015 [45]	China	343	1 year	Beihai (I-deficient areas) (*n* = 103)			41	Beihai (I-deficient areas) (*n* = 113)			51		
				Yangcheng and Jiajiazhuang (I-sufficient areas) (*n* = 91)			346	Yangcheng and Jiajiazhuang (I-sufficient areas) (*n* = 98)			282		
				Pingyao and Jicun (I-excess areas) (*n* = 99)			942	Pingyao and Jicun (I-excess areas) (*n* = 125)			823		
Liu et al., 2020 [46]	China	218	0–12 months	Suitable water iodine content areas (*n* = 97)			312	Suitable water iodine content areas (*n* = 97)			284		
				High water iodine content areas (*n* = 121)			1006	High water iodine content areas (*n* = 121)			823		
Mobasseri et al., 2014 [47]	Azerbaijan	106	NS			58				142			
Nazeri et al., 2018 [48]	Iran	124	<3 months			100				78			
Ordookhani et al., 2007 [50]	Iran	48	37 to 42 weeks			148				107			
Osei et al., 2016 [51]	South Africa	100	2–4 months			179				118			
Osei et al., 2016 [52]	South Africa	371	6 months			180				128			
Pal et al., 2017 [53]	India	128	1–3 months			230				185			
Pearce et al., 2007 [54]	United States	57	48 days ^1^			155				114			
Petersen et al., 2020 [55]	Iceland	60	25 weeks			84				152			
Pongpaew et al., 1999 [56]	Thailand	75	233 days ^1^			51				90			
Prpić et al., 2021 [57]	Croatia	133	2–96 weeks			121				75			
Samson et al., 2021 [58]	Hungary	100	NS			188				49			
Stinca et al., 2017 [60]	Morocco	239	≤8 weeks			42				35			
Vermiglio et al., 1992 [62]	Italy	27	5–7 days	Endemic group (*n* = 11)			33 ^2^	Endemic group (*n* = 11)			12 ^2^		Small sample size
				Control group (*n* = 16)			43 ^2^	Control group (*n* = 16)			63 ^2^		
Wang et al., 2018 [63]	China	106	4–12 weeks	4 weeks			222 ^2^	4 weeks			152		
				8 weeks			175 ^2^	8 weeks			112		
				12 weeks			148 ^2^	12 weeks			109		
Wang et al., 2009 [64]	China	100	0–1 year			163				136			
Yan et al., 2005 [65]	China	2554	0–2 year	Urban			136	Urban			189		Huge study in 11 provinces of China
				Rural			158	Rural			192		
***Interventional study***													
Bouhouch et al., 2014 [66]	Morocco	241	0–9 months		**Indirect infant supplementation**		**Direct infant supplementation**		**Indirect infant supplementation**		**Direct infant supplementation**	One dose of 400 mg iodine as oral iodised oil soon after delivery	
				Baseline	41		43	Baseline	37		30		
				3-month	61		33	3-month	58		34		
				6-month	49		36	6-month	67		44		
				9-month	39		26	9-month	58		39		
Eriksen et al., 2020 [67]	The Gambia	219	12 weeks	Baseline (<20 weeks of gestation)			-	Baseline (<20 weeks of gestation)			51		A daily supplement of multiple micronutrient containing 300 µg of iodine was taken starting from baseline (<20 weeks of gestation) until delivery. Only both BMIC and UIC data of lactating women at 12 weeks were available.
				12 weeks			51	12 weeks			39		
Gebreegziabher et al., 2017 [68]	Ethiopia	101	6 month			**Capsule group**	**I-salt group**			**Capsule group**	**I-salt group**		225 μg iodine as potassium iodide capsule daily for 6 months or 450 g of appropriately iodized salt (30–40 μg I as KIO3/g of salt) weekly for household consumption for 6 months
				Baseline		149	157	Baseline		136	95		
				6 months		104	111	6 months		150	110		
Nazeri et al., 2017 [69]	Iran	84	1 month			**Iodine fortified milk group (*n* = 40)**	**Control group (*n* = 40)**			**Iodine fortified milk group (*n* = 40)**	**Control group (*n* = 40)**		200 mL iodine fortified milk of which provided 150 µg iodine/day, started at the sixth day postpartum and lasted for four weeks
				3–5 days (baseline)		176	215	3–5 days (baseline)		70	97		
				7 days		191	176	7 days		119	51		
				10 days		217	162	10 days		131	103		
				14 days		242	160	14 days		123	48		
				1 month		210	142	1 month		104	41		
Stoutjesdijk et al., 2018 [70]	Netherlands	36	4 weeks	20 gestational weeks (baseline)		-		20 gestational weeks (baseline)		102			Multivitamin supplement containing 150 μg/day of iodine were given during 20 gestational weeks
				4 weeks		152		4 weeks		112			
Sukkhojaiwaratkul et al., 2014 [61]	Thailand	87	2 months	3rd trimesters (baseline)		-		3rd trimesters (baseline)		204			Multivitamin supplement containing 200 μg/day of iodine were given during 2-month postpartum
				Total		91		Total		138			
				Iodine-supplemented (200 µg)		109		Iodine-supplemented (200 µg)		199			
				non-supplemented		70		non-supplemented		120			

^1^ Median used unless mean reported, ^2^ mean (µg/L), ^3^ mean (µg/g), NS, not stated. References no. [20] and [45] were counted more than once in Section 3.1 because they reported findings in both iodine deficient and sufficient populations.

**Table 4:** BMIC of lactating women and UIC of infants.

Author, Year	Country	Sample Size of Infants	Time of Postpartum (Days/Weeks/Months)	BMIC ^1^ (µg/L)			UIC ^1^ (µg/L)			Comments	
***Observational study***											
Aakre et al., 2016 [22]	Algeria	289	31.4 days ^1^	479			722				
Anderson et al., 2010 [24]	Switzerland	875	6–12 months	49			82				
Budak et al., 2009 [28]	Turkey	35	5–28 days	33 ^2^			100				
Chen et al., 2020 [30]	China	634	24 weeks	165			216				
Costeira et al., 2009 [32]	Portugal	142	3 months	3 days		95	3 days	65			
				3 months		70	3 months	96			
Chung et al., 2009 [31]	Korea	31	6 weeks	3rd week		1153	3rd week	1651		Small sample size	
				6th week		822	6th week	1832			
de Lima et al., 2013 [33]	Brazil	33	≤6 months	206			293			Small sample size	
Dold et al., 2017 [20]	China, Philippines and Croatia	866	3 months	China (*n* = 298)		170	China (*n* = 298)	278			
				Philippines (*n* = 281)		185	Philippines (*n* = 281)	352			
				Croatia (*n* = 73)		124	Croatia (*n* = 73)	239			
Dumrongwongsiri et al., 2018 [34]	Thailand	71	NS	255			282				
Huynh et al., 2017 [38]	Australia	696	3 months	127			198				
Isiklar Ozberk et al., 2018 [39]	Turkey	107	NS	97			95				
Jin et al., 2021 [40]	New Zealand	87	3 months	69			115				
Kart et al., 2021 [41]	Turkey	334	4–6 days	138			142				
Kurtoglu et al., 2004 [43]	Turkey	70	5 days	73			24				
Liu et al., 2015 [45]	China	343	1 year	Beihai (I-deficient areas) (*n* = 103)		41	Beihai (I-deficient areas) (*n* = 28)	65			
				Yangcheng and Jiajiazhuang (I-sufficient areas) (*n* = 91)		346	Yangcheng and Jiajiazhuang (I-sufficient areas) (*n* = 90)	427			
				Pingyao and Jicun (I-excess areas) (*n* = 99)		942	Pingyao and Jicun (I-excess areas) (*n* = 124)	1222			
Liu et al., 2020 [46]	China	218	0–12 months	Suitable water iodine content areas (*n* = 97)		312	Suitable water iodine content areas (*n* = 97)	427			
				High water iodine content areas (*n* = 121)		1006	High water iodine content areas (*n* = 121)	1222			
Mobasseri et al., 2014 [47]	Azerbaijan	106	NS	58			307				
Nazeri et al., 2018 [48]	Iran	124	<3 months	100			183				
Ordookhani et al., 2007 [50]	Iran	27	37 to 42 weeks	148			271			Small sample size	
Osei et al., 2016 [51]	South Africa	100	2–4 months	179			373				
Osei et al., 2016 [52]	South Africa	386	6 months	180			345				
Pal et al., 2017 [53]	India	128	1–3 months	230			250				
Prpić et al., 2021 [57]	Croatia	133	2–96 weeks	121			2–26 weeks (*n* = 101)	234			
							27–96 weeks (*n* = 32)	209			
Skeaff et al., 2005 [59]	New Zealand	230	6–24-month	22			67				
Stinca et al., 2017 [60]	Morocco	239	≤8 weeks	42			73				
Vermiglio et al., 1992 [62]	Italy	27	5–7 days	Endemic group (*n* = 11)		33 ^2^	Endemic group (*n* = 11)	34 ^2^		Small sample size	
				Control group (*n* = 16)		43 ^2^	Control group (*n* = 16)	43 ^2^			
Wang et al., 2018 [63]	China	106	4–12 weeks	4-week		222 ^2^	4-week	251			
				8-week		175 ^2^	8-week	183			
				12-week		148 ^2^	12-week	164			
Wang et al., 2009 [64]	China	61	0–1 year	163			233				
Yan et al., 2005 [65]	China	2537	0–2 years	Urban		136	Urban	236		Huge study in 11 provinces of China	
				Rural		158	Rural	247			
***Interventional study***											
Bouhouch et al., 2014 [66]	Morocco	241	0–9 months		**Indirect infant supplementation**	**Direct infant supplementation**		**Indirect infant supplementation**	**Direct infant supplementation**	One dose of 400 mg iodine as oral iodised oil soon after delivery	
				Baseline	41	43	Baseline	73	74		
				3-month	61	33	3-month	132	99		
				6-month	49	36	6-month	142	122		
				9-month	39	26	9-month	97	90		
Gebreegziabher et al., 2017 [68]	Ethiopia	101	6 months		**Capsule group**	**I-salt group**		**Capsule group**	**I-salt group**	225 μg iodine as potassium iodide capsule daily for 6 months or 450 g of appropriately iodized salt (30–40 μg I as KIO3/g of salt) weekly for household consumption for 6 months	
				Baseline	149	157	Baseline	234	193		
				6-month	104	111	6-month	254	195		
Gutierrez-Repiso et al., 2014 [36]	Spain	88	NS	**Control group (*n* = 21)**	**109**		**Control group (*n* = 21)**	112		300 µg of iodide (in the form of KI) were given from the first trimester of pregnancy (300 group)	
				300 group (*n* = 67) (300 µg)	178		300 group (*n* = 67) (300 µg)	215			
Kirk et al., 2012 [42]	United States	13	1–8 months	Pre supplementation	53 ^2^		Pre supplementation	239		Small sample size	
				PM supplementation	57 ^2^		PM supplementation	379			
				AM supplementation	57 ^2^		AM supplementation	324			
Nazeri et al., 2017 [69]	Iran	84	1 month		**Iodine fortified milk group (*n* = 40)**	**Control group (*n* = 40)**		**Iodine fortified milk group (*n* = 40)**		**Control group (*n* = 40)**	200 mL iodine fortified milk of which provided 150 µg iodine/day, started at the sixth day postpartum and lasted for four weeks
				3–5 days (baseline)	176	215	3–5 days (baseline)	231		193	
				7 days	191	176	7 days	169		120	
				10 days	217	162	10 days	219		138	
				14 days	242	160	14 days	194		116	
				1 month	210	142	1 month	230		110	
Nøhr et al., 1994 [49]	Denmark	147	5 days	Baseline	34		Baseline	32		Vitamin-mineral preparations containing iodine (with a declared iodine content of 150 µg/tablet).	
				Not received iodine supplementation group (*n* = 94)	34		Not received iodine supplementation group (*n* = 94)	32			
				Tablets containing iodine group (*n* = 53)	57		Tablets containing iodine group (*n* = 53)	61			

^1^ Median used unless mean reported, ^2^ mean, NS, not stated. Reference no. [45] was counted more than once in Section 3.2 because it reported findings in both iodine deficient and sufficient populations.

## Data Availability

Not applicable.

## Supplementary Materials

The following supporting information can be downloaded at: https://www.mdpi.com/article/10.3390/nu14091691/s1, Table S1. The Jadad scale for assessment of study quality for intervention studies (Jadad et al., 1996), Table S2. The Jadad scores of included studies, Table S3. Assessment of quality for a cohort study; adapted from the Newcastle-Ottawa scale. Stars were awarded if the criteria shown in italics were met. The number of stars awarded are indicated at the end of each statement. Maximum of 13 stars*.
