# Supplementary material for: Breast Milk Iodine Concentration (BMIC) as a Biomarker of Iodine Status in Lactating Women and Children <2 Years of Age: A Systematic Review
Source: Nutrients. 2022 Apr 19;14(9):1691. doi: 10.3390/nu14091691 (PMC9104537; doi:10.3390/nu14091691)
Supplement: Supplementary file 1 [file nutrients-14-01691-s001.zip › nutrients-1617259-supplementary.pdf]

Supplementary Table S1. The Jadad scale for assessment of study quality for intervention studies (Jadad et al., 1996)

Item	Description	Yes	No
1	Was the study randomised?	Score 1	Score 0
2	Was the method of randomisation described, and appropriate?	Score 1	Score -1
3	Was the study performed double-blind?	Score 1	Score 0
4	Was the method of blinding described and appropriate?	Score 1	Score -1
5	Were withdrawals and drop outs of subjects described?	Score 1	Score 0
	Total		

Supplementary Table S2. The Jadad scores of included studies

Reference	Described as randomised? (+1)	Randomization described and appropriate? (-1, +1)	Described as double blind? (+1)	Double blinding described and appropriate? (-1, +1)	Number and reasons for withdrawals described? (+1)	Total Jadad score (max 5)

Supplementary Table S3. Assessment of quality for a cohort study; adapted from the Newcastle-Ottawa scale. Stars were awarded if the criteria shown in italics were met. The number of stars awarded are indicated at the end of each statement. Maximum of 13 stars*.

Selection

1) Representativeness of the exposed cohort (max 2 stars*)

*a) truly representative of offspring (infant/children) born to mothers in developed and developing countries ***

*b) somewhat representative of offspring (infant/children) born to mothers in developed and developing countries **

c) selected group of offspring, for example only certain socioeconomic groups/areas

d) no description of the derivation of the cohort

2) Ascertainment of exposure (max 2 stars*)

*a) measurement by a trained health professional/researcher ***

*b) structured interview **

c) written self report (questionnaire etc.)

d) other/no description

3) Demonstration that outcome of interest was not present at start of study (max 1 star*)

*a) yes **

b) no

Comparability

1) Comparability of cohorts on the basis of the design or analysis (max 4 stars*)

*a) study states that physical factors have been controlled for in statistical analysis (all of the following: age and sex) **

*b) study states that maternal factors have been controlled for in statistical analysis (all of the following: breast milk concentrations, age at delivery, parity, weight, height) **

*c) study states that socioeconomic status has been controlled for in statistical analysis (both education and income) **

*d) study states that lifestyle factors have been controlled for in statistical analysis (all of the following: smoking, alcohol) **

Assessment

1) Assessment of outcome (max 1 star*)

*a) independent assessment by trained health professional/researcher **

b) record linkage

c) self report

d) no description

2) Was the lactation follow-up period or the child aged ≤ 2 years (max 1 star*)

*a) yes**

b) no

3) Adequacy of follow up of cohorts (max 2 stars*)

*a) complete follow up - all subjects accounted for ***

*b) subjects lost to follow up unlikely to introduce bias: number lost ≤ 20 %, or description provided of those lost suggesting no difference from those followed up **

c) follow up rate < 80% and no description of those lost

d) no statement
